# Western Journal of Medicine and Surgery

**Published:** 1850-12

**Authors:** 


### THE MEDICAL EXAMINER.

PHILADELPHIA, DECEMBER, 1850.

### WESTERN JOURNAL OF MEDICINE AND SURGERY.

We have received and read the reply of Dr. T. S. Bell, to "Friendly Hints to a Reviewer." As both sides have now been heard, and have pretty well exhausted the subject, and as we do not conceive that any good can flow from its further discussion, we have preferred to put an end to the matter so far as our pages are concerned.

